# Phenotyping Latin American Open-Pollinated Varieties of Popcorn for Environments with Low Water Availability

**DOI:** 10.3390/plants10061211

**Published:** 2021-06-15

**Authors:** Talles de Oliveira Santos, Antônio Teixeira do Amaral Junior, Rosimeire Barboza Bispo, Valter Jário de Lima, Samuel Henrique Kamphorst, Jhean Torres Leite, Divino Rosa dos Santos Júnior, Pedro Henrique Araújo Diniz Santos, Uéliton Alves de Oliveira, Kátia Fabiane Medeiros Schmitt, Eliemar Campostrini, Monique Moreira Moulin, Alexandre Pio Viana, Geraldo de Amaral Gravina, Caio Cezar Guedes Corrêa, Gabriel Moreno Bernardo Gonçalves

**Affiliations:** 1 Laboratory of Genetics and Plant Breeding, Universidade Estadual do Norte Fluminense Darcy Ribeiro (UENF), Campos dos Goytacazes 28013-602, RJ, Brazil; rosimeirebarboza1@hotmail.com (R.B.B.); valter_jario@hotmail.com (V.J.d.L.); samuelkampho@hotmail.com (S.H.K.); torresjhean@gmail.com (J.T.L.); juniorifagro@gmail.com (D.R.d.S.J.); phsantos2004@yahoo.com.br (P.H.A.D.S.); uelitonalves2011@hotmail.com (U.A.d.O.); kmedeirosschmitt@gmail.com (K.F.M.S.); campostenator@gmail.com (E.C.); pirapora@uenf.br (A.P.V.); caiocagronomo@gmail.com (C.C.G.C.); gabriel.agrobio@gmail.com (G.M.B.G.); 2 Laboratory of Genetics and Molecular Biology, Instituto Federal do Espírito Santo—Campus de Alegre (IFES), Alegre 29500-000, ES, Brazil; mmmoulin@ifes.edu.br; 3 Laboratory of Agricultural Engineering, Universidade Estadual do Norte Fluminense Darcy Ribeiro (UENF), Campos dos Goytacazes 28013-602, RJ, Brazil; gravina@uenf.br

**Keywords:** drought, genetic resources, gt biplot, multivariate analysis, water stress

## Abstract

Drought is a common abiotic stress in tropical and subtropical regions that limits the growth and development of agricultural crops, mainly impacting grain yield. Acting through plant breeding is the most viable alternative for obtaining genotypes more tolerant of environments with stress. This work aims to select popcorn genotypes for environments with drought and to identify discriminating traits for the evaluation of drought tolerance in popcorn germplasm. Fifteen Latin American populations of popcorn were evaluated in water stress (WS) and well-watered (WW) conditions. The evaluated traits were based in morpho-agronomic, physiological and radicular descriptors. Data were submitted to individual and joint ANOVA and GT Biplot analysis. Variability was detected between populations for all traits in both conditions. The drought caused a reduction of 30.61% and 3.5% in grain yield and popping expansion, respectively. Based in GT biplot analysis, 880POP was the most stable in WS and WW, being indicated as a promising population for cultivation in environments with water limitation. This study is going to allow the establishment of a collection of great importance to maize germplasm and to provide information to facilitate the process of selection in breeding programs focused on drought tolerance.

## 1. Introduction

Water stress is a common abiotic stress in tropical and subtropical regions, including Brazil [1], and periods of drought have become frequent due to the global effects of climate change [2]. Therefore, by 2050, an estimated 2.3 billion people will suffer from water shortages [3]. In addition to the scarcity of rainfall, it is pointed out that, of all water destined for human activities, about 70% is demanded by agriculture [4]. According to data from FAO [5], Brazil stands out among the ten countries with the largest irrigated areas in the world.

Abiotic stresses have a negative impact on plant growth and development, preventing the expression of the genetic potential of agricultural crops [6]. Numerous restrictions to the metabolic processes of plants occur during a period of drought, which is caused by high evaporative demand and a limited supply of water in the soil, which, by restricting photosynthetic activity, affects plant growth and development [7]. Water stress is identified as the biggest cause of productivity losses in agricultural crops [8,9,10] and, among them, popcorn (*Zea mays everta*) [10,11].

When it comes to popcorn, a highly appreciated and economically attractive crop, research aiming at the selection of drought-tolerant genotypes is still incipient. In Brazil, the state of Mato Grosso is the largest producer of this cereal, having reached the record mark of 268,402 thousand tons produced in a cultivated area of 60,017 hectares in the agricultural year of 2018. The area for production for the year of 2019 expanded to 66,986 hectares, which represents an increase of 11.61% [12].

The selection of superior genotypes for drought conditions is the most economically viable strategy to minimize the effects of drought [13,14]. However, the development of cultivars for adverse water supply conditions depends on accurate characterization and the selection of traits capable of discriminating the tolerance of the studied genotypes, which is only successfully achieved when there is genetic variability. In this perspective, we highlight the open-pollinated varieties that, despite being less productive than hybrids, have a broader genetic base and, therefore, can be used as important gene sources for tolerance to drought stress [15]. Furthermore, the possibility of registering these populations per se for indication to small producers is not ruled out, since the genetic structure of open-pollination populations gives greater tolerance to stresses in general and allows the use of the same seeds in future cycles. In this sense, registering these materials guarantees the producer less dependence on the acquisition of hybrids.

In this sense, the characterization of germplasm for drought tolerance is necessary from the point of view of popcorn breeding, since in Brazil there is only one hybrid with drought tolerance registered for the species–registered at the Ministry of Agriculture, Livestock and Supply under the register number 46965 (Lima et al. 2021, in press). In this regard, secondary traits with high correlation with grain yield can be used for indirect selection or composition of selection indexes, seeking to make the improvement process for tolerance to water stress effective [16].

Therefore, the development of this study was considered opportune, in which popcorn populations from different Latin American regions were used, with the following objectives: (i) to identify discriminating traits for the reliable evaluation of germplasm that may be tolerant to drought; and (ii) select genotypes for environments with low water availability, based on morphological, physiological and root descriptors.

## 2. Results

### 2.1. Populations’ Performance in Well-Irrigated and Water Stress Conditions

Concerning the morpho-agronomic, physiological and root traits, analysis of variance (ANOVA) resulted in the detection of significant genotypic variation between the 15 genotypes in WW and WS conditions, statistically proven by Test F (*p* < 0.01) except for relative chlorophyll content (SPAD) and crown root angle (CRA) in WW and number of rows of grains (NRG) in WS. In both water conditions, the coefficients of variation did not exceed the value of 20%, pointing to a satisfactory level of experimental accuracy (Table 1).

For SPAD, the reduction caused by the drought was 15.80%. In WW the average value of this trait was 33.25 while in WS it was 28.02. The water limitation did not affect the plant height (PH) in an expressive way, and the reduction in trait was of 9.95%. The tassel length (TL) decreased by 0.72%. In WW conditions, this trait had an average value of 12.04 cm, while in WS conditions the average value was 11.95 cm. The number of tassel branches (NTB) showed a significant reduction. In the WW, the tassels had an average of 19 branches, while in WS this value was an average of 16, which means a 20% reduction (Table 1).

The yield-related traits ear length (EL), number of rows of grains (NRG), number of grains per row (NGR) and one hundred grain weight (100GW) under WS conditions were reduced by 11.67%, 4.51%, 15.24% and 5.76%, respectively. In the WW environment, the mean values for these traits were 12.79 cm (EL), 13.24 (NRG), 27.41 (NGR) and 15.99 g (100GW), compared to 11.30 cm, 12.64, 23.24 and 15.07 g, in that order, in WS. The reduction in GY due to water limitation was 30.61%, given the average of 1862.62 kg ha^−1^ in WS and 2684.28 kg ha^−1^ in the WW condition. The reduction of 3.50% in PE was not significant. In WS the average value of this trait was 20.14 mL g^−1^ and 20.87 mL g^−1^ in the WW condition.

The traits associated with the root architecture-support root angle (SRA), crown root angle (CRA) and (number of crown roots) NCR-suffered a reduction in WS of 0.60%, 1.50% and 1.20%, respectively. For number of support roots (NSR) and density of crown roots (DCR), the water deficit provided increases of 1.90% and 20.30%, respectively. Of these, the greatest effect of stress was on DCR.

### 2.2. GT Biplot Analysis

**Figure 1:**
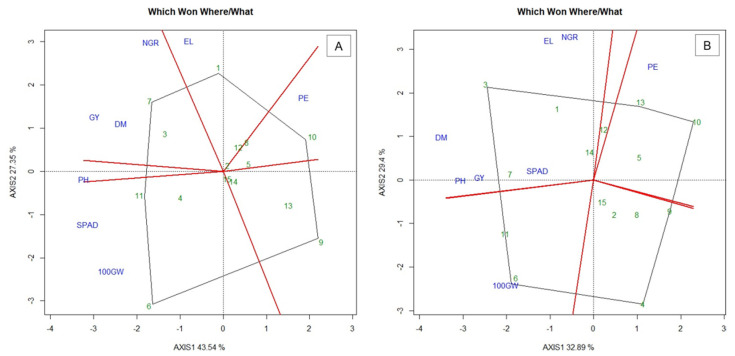
Biplot “which-won-where” graph for (**A**) water stress (WS) and; (**B**) well-watered (WW) conditions. 100GW—100 grains wight; GY—grain yield; PH—plant height; DM—shoot dry matter; NGR—Number of grains per row; EL—ear length; PE—popping expansion; SPAD—relative chlorophyll content; (-) signal indicating negative values.

In the WS condition, the first two Principal Components (PCs) explained 70.89% of the total variation in traits between the evaluated genotypes (Figure 1A). In the WW condition, the first two PCs explained 62.29% of this variation (Figure 1B).

In the “which won where/what” graph of the biplot analysis in the WS, six distinct groups were formed with the studied traits (Figure 1A). According to Yan [17], in this graph, groups of traits are formed by traits between the perpendicular lines and these groups are used to identify the genotypes with the best prominence for these traits. Thus, the genotypes located at the vertices of the polygon are far from the center of origin of the graph, indicating their best performance for the group of characteristics [17,18,19]. In contrast, the genotypes within the polygon are those that are least responsive to the characteristics studied. The first group included only the trait ear length (EL). Similarly, the second group was formed only by popping expansion (PE), while the third group was formed by 100-grains weight (100GW) and SPAD. The fourth group was formed only by plant height (PH), while the fifth group was formed by grain yield (GY), dry matter (DM) and number of grains per row (NGR).

The genotype 288POP (1), which appeared at the vertex of the first group, stood out for presenting greater average value for EL. Group two presented the ISLA (10) genotype at its vertex, which stood out for exhibiting the highest estimate for PE. In group four, BOYA462 (6) and PARA172 (11) genotypes stood out, as they presented higher values for 100GW and SPAD. In group five, there was no vertex formation of the biplot polygon, while in group six the BOZM260 (7) genotype stood out, being the population with the highest GY, DM and NGR (Figure 1A). In WW, four groups were formed (Figure 1B). The first group included DM, PH, GY, EL, NGR and SPAD. Group two, as in WS, was formed only by the PE trait. The group three, on the other hand, did not have any traits and group four was formed by the 100GW.

The population 880POP (3) stood out for the first group of traits, as it constituted the vertex of the polygon. Thus, it gathered higher values of DM, PH, GY, EL, NGR and SPAD. For group two, as in WS conditions, the ISLA (10) genotype formed the vertex of the polygon, once it presented the highest value for PE, followed by UNB2-C6 (13)—although less accentuated, due to its estimate of 25.11 g mL^−1^ for PE—considered the tenth average value in the ranking of the magnitudes of PE. The third group, despite highlighting a genotype, does not indicate an efficient trait separating it from the others. In group four, the population BOYA462 (6) stood out, with higher 100GW.

**Figure 2:**
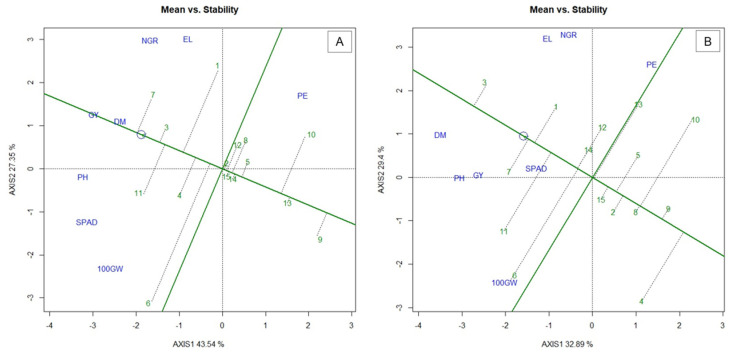
Biplot “mean vs. stability” graph for (**A**) water stress (WS) and; (**B**) well-watered (WW) conditions. 100GW—100 grains wight; GY—grain yield; PH—plant height; DM—shoot dry matter; NGR—Number of grains per row; EL—ear length; PE—popping expansion; SPAD—relative chlorophyll content; (-) signal indicating negative values.

In the biplot “means vs. stability” analysis, performance is defined as the longest vector of principal component 1 (PC1), represented by the one with the arrow indicating the ideal genotype, which is located in the center of the concentric circle (Figure 2). Thus, genotypes located to the left of the center of origin are those with higher performance, while those located on the right, have lower performance. Stability, on the other hand, can be observed through principal component 2 (PC2), because the smaller the projection (dashed line) of a given genotype, closer it becomes to the center of the biplot, presenting greater stability for the evaluated traits. Thus, ideal genotypes must have a high PC1 value (high yield capacity) and low PC2 value (high stability) [20].

In the WS condition, the genotypes 288POP (1), 880POP (3), ARZM 13050 (4), BOYA462 (6), BOZM260 (7) and PARA172 (11) presented values above the general average for the studied traits (Figure 2A). In contrast, nine genotypes showed values below the average, being: 574POP, BARÃO-UFV (5), BRS Angela (8), CHZM13134 (9), ISLA (10), UNB2-C0 (12), UNB2-C6 (13), UNB2-C8 (14) and URUG298A (15). In the WS, only the population BOZM260 (7) was found to be ideal, having high performance compared to the general average of the traits and greater stability. However, the population 880POP (3), even though it does not stand out as the closest to the ideal, also shows considerable stability in WS conditions.

In WW, it was observed that seven genotypes—288POP (1), 880POP (3), BOYA462 (6), BOZM260 (7), PARA172 (11), UNB2-C0 (12) and UNB2-C8 (14)—showed values above the general mean for the evaluated traits. The population 880POP was considered the one with the highest mean value for the evaluated traits, as well as the one with the highest stability (Figure 2B).

**Figure 3:**
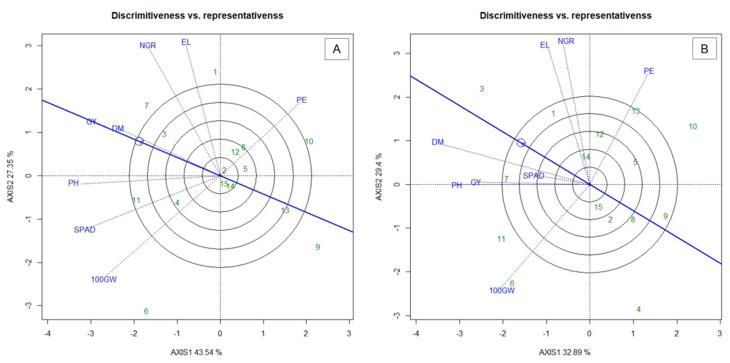
Biplot “discriminativeness vs. representativeness” graph for (**A**) water stress (WS) and; (**B**) well-watered (WW) conditions. 100GW—100 grains wight; GY—grain yield; PH—plant height; DM—shoot dry matter; NGR—Number of grains per row; EL—ear length; PE—popping expansion; SPAD—relative chlorophyll content; (-) signal indicating negative values.

In the “discriminativeness vs. representativeness” analysis (Figure 3), the ability of a trait to discriminate a genotype is highlighted by the size of the vector (dashed line). The longer the vector, the more discriminating the trait is [21]. The most representative traits are those that form the smallest angles with the line that presents the circle formed with the arrow [22]. Considering that the ideal trait should be able to discriminate the genotypes and represent the other traits, it would present high PC1 (greater discriminatory capacity) and low PC2 (greater representativeness) [22].

In this sense, in WS conditions, PH, SPAD, GY and DM (Figure 3A) stood out as potential traits for greater discrimination and representativeness. PE in WS was not representative, but has a discriminant potential. In the WW condition, the traits DM, GY and PH were the most discriminating and representative, behaving similarly to the WS condition (Figure 3B). The characteristics 100GW, EL and NGR provided high discrimination, but were not very representative. In turn, SPAD was not very discriminating and not representative.

**Figure 4:**
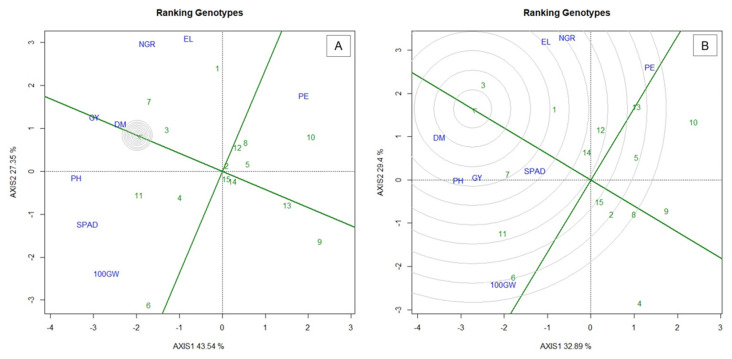
Biplot “ranking genotypes” graph for (**A**) water stress (WS) and; (**B**) well-watered (WW) conditions. 100GW—100 grains wight; GY—grain yield; PH—plant height; DM—shoot dry matter; NGR—Number of grains per row; EL—ear length; PE—popping expansion; SPAD—relative chlorophyll content; (-) signal indicating negative values.

The “ranking genotypes” graph of the biplot analysis shows the classification of genotypes based on the ideal genotype, or ideotype (Figure 4). An ideal genotype is a hypothetical genotype that has the highest yield and stability, which leans on the biplot graph to the positive end of the axis and has the shortest vertical distance from it, being indicated as the one closest to the concentric circle [17]. In this study, based in analysis of ranking genotypes (Figure 4), the populations that came closest to the ideotype were, in this order, for WS (Figure 4A) and WW (Figure 4B): BOZM260 (7), 880POP (3) and 288POP (1); and 880POP (3), BOZM260 (7) and 288POP (1).

## 3. Discussion

The reduction caused in SPAD with the imposition of drought can be explained by the fact that in plants, when in low water availability, stomatal closure occurs and, consequently, the induction of the formation of reactive oxygen species (ROS) that inhibit enzymes in the Calvin cycle [23] and degrade fundamental cell structures, as well as important photosynthetic compounds, such as chlorophyll [24], also occurs.

In a study on common maize inbred lines and hybrids, Cairns et al. [25] when evaluating leaf chlorophyll content under water stress conditions, found reductions of 29.33% (37.5 in WW and 25.6 in WS) and 17.49% (46.3 in WW and 38.2 in WS), respectively. The reduction in greater proportions of the lines was expected, since there is great depression due to inbreeding in plants with this genetic structure, whereas in hybrids the percentage reduction is smaller, considering the positive effect of heterosis. It is noticed that the results of studies with hybrids demonstrate that even in the face of the reduction in SPAD caused by WS, the values in stress conditions are higher than those of the populations in our work. However, it is important to note that populations show lower percentage losses. However, it is noteworthy that the smallest decrease in SPAD in our study may be related to the broad genetic basis of the populations, which, being formed by heterogeneous individuals, have greater genotypic variability and, therefore, feel the impacts of environmental changes less. 

Regarding the low reduction of the plant height, the imposition of water stress in the pre-flowering period may be related to this low reduction, given that plants in this period are close to the end of vegetative development, being little affected by water limiting conditions; the growth would be affected if applied in seedling to V6 stages [26,27]. The tassel length did not show a significant reduction; however, the number of branches of the tassel showed a significant reduction. It is reported in the literature the existence of a dominance in relation to the development of ears and tassels and that the difference of this dominance in plants varies between genotypes [28]. In maize, this difference is related to monoicy and protandry, which favors the development of male inflorescence despite of female, ensuring the production of pollen before the complete maturation of female reproductive organs [28,29]. Despite this difference in conditions of water stress, some studies report the important relationship between the smallest size and the largest number of branches in the tassel.

Some authors support the hypothesis that the association between detasseling and increased productivity may be related to competition between ears and tassel for water resources [30,31,32]. Other studies associate productivity increase in plants with reduced tassels to the low competition for light between this structure and the primary leaves, which guarantee photoassimilates for the grain filling process [33,34]. As an example, Monneveux et al. [28] point to an increase in the productivity of maize hybrids in conditions of water stress when the plants have lower numbers of these structures.

It can be inferred that the reduction in GY of 30.61% (from 2684.28 kg ha^−1^ in WW to 1862.62 kg ha^−1^ in WS) occurred mainly due to the impact of water stress on number of grains per row and the ear length, which expressed a decrease of 15.24 and 11.67%, respectively. In common maize, the condition of drought in the soil can compromise up to 60% of the grain yield when the stress coincides with the pre-flowering and grain filling phases, a critical period for the crop [35,36]. The reduction of these traits (GY, EL and NGR) can be associated with the fact that water stress may shorten the period of grain filling, decrease photosynthetic activity, accelerate ABA-mediated leaf senescence and reduce the activity of enzymes involved in sucrose metabolism and starch synthesis, which altogether can contribute to a lower grain weight [37,38,39].

The popping expansion is considered an important quality trait for the popcorn trade, and it is expressed by the ratio of popcorn volume by the weight of grains. PE values between 25 and 30 are considered to be regular, values between 30 and 35 are considered as good and populations with PE above 35 are classified as excellent. This trait had a low effect of the water stress (3.50%). One hypothesis for this result is the wide genetic basis of open-pollinated varieties, which suffer less from environmental stresses, guaranteeing them greater phenotypic stability. In other works with popcorn performed by Kamphorst et al. [40] and Lima et al. [12], is possible to see that PE presented higher reductions of 8.76% and 9.08%, respectively, between the WW and WS conditions. However, it is necessary to take into account that these losses for GY and PE are more accentuated since the authors are using inbred lines, i.e., materials suffering the effects of inbreeding, being more sensitive to low water availability in soil.

The morphological adaptation of the root angle (SRA and CRA) has already been described by York et al. [41] who, when analyzing the root system of maize genotypes under water stress conditions, proposed the root ideotype ‘Steep, Cheap and Deep’ for drought conditions. This is because genotypes with a steep root system are able to fetch water from profiles farther from the soil. Root phenotypes with a smaller number and larger size of cortical cells reduce the metabolic cost of soil exploration. Subsequently, Gao and Lynch [42] added the reduced number of crown roots (NCR) as an element of this ideotype, considering that based on complementary studies, this characteristic was the one that allowed the deepening of the root system. The crown roots are responsible for the acquisition of a good part of the water and mineral resources during vegetative growth and it is shown to be important during the reproductive development, period in which the water stress is more critical [42,43]. In the present study, the reduction in NCR suggests a root adaptation of the studied populations to the conditions of low water availability in soil. In addition, the increase in DCR (20.30%) may be associated with a greater presence of root hair, which guarantees a larger surface for water absorption in the event of a dry condition.

According to Silva [44], the principal component analysis improves the interpretation of the data, since it contains as much information as possible in a smaller number of PCs. For the biplot analysis, according to Yang et al. [45], the first two PCs must explain more than 60% of the data variation in order to have a reliable interpretation of the results. In the present work, this premise was fulfilled both in the WS and WW conditions, which allowed perform the safe multivariate selection of genotypes that presented the best performance for the studied characters.

In the present study, the genotypic superiority of 288POP, ISLA, BOYA462 and BOZM260 encourages breeding programs aimed at obtaining superior genotypes of popcorn for adaptation to environments with low water availability. It is a group of genotypes that stand out for traits of high interest for the selection of genotypes for greater efficiency in conditions of water limitation, namely: PE, 100GW, GY, DM and NGR; therefore, they are believed to have alleles that favor tolerance to water stress or efficiency in the use of water. 

Concerning the consonance in the grouping of the BOYA462 and ISLA genotypes for 100GW and PE, observed in both conditions, Miranda et al. [46] highlight that it is more likely to obtain an increase in grain productivity in Brazilian materials from the use of local populations, having recommended the use of tropical germplasm for this purpose; on the other hand, increases in PE are possible through the use of temperate climate germplasm. It is not by chance that, in the present study, greater emphasis on PE occurred with the genotype that has alleles for temperate/tropical adaptation—in the ISLA case, in both WS and WW conditions, while the greater expectation of increase in grain yield fell to only subtropical adaptation genotypes, in this case, 574POP and 880POP, in WS and WW, respectively. This suggests a good adaptation of the temperate climatic adaptation materials to the conditions studied for the PE characteristic. In this sense, the inclusion of these materials in studies with popcorn becomes an incentive to obtain superior materials. However, there are still few studies that include materials with different climatic adaptations. Thus, the constitution of a compound containing the ISLA, 574POP and 880POP genotypes seems to be the most advantageous alternative for the extraction of lines and, consequently, obtaining hybrids and, even, for the implementation of recurrent intrapopulation selection for Brazilian conditions.

Breeding programs seek to balance the selection of more stable and productive genotypes [47]; thus, the 880APOP genotype would be ideal in WS conditions since, in the water limitation scenario, it expressed more stable behavior for the evaluated traits, whereas even being considered the one with the highest yield, the BOZM260 genotype would not express stability in the same 880POP level. Comparing the two conditions, it is noted that the 880POP genotype maintains good phenotypic stability under WS. Plant breeding aiming at phenotypic stability of traits associated with productivity has become an important tool in the launch of cultivars adapted to adverse conditions [16], especially for regions where water supply is limited [48].

For popcorn, the PE, which is the main quality trait of the grains, should not be discarded [49]. In this regard, the inclusion of ISLA, 574POP and 880POP for the formation of compost is of fundamental importance, because ISLA has greater phenotypic expression for PE. Although there is a discrimination of ISLA due to its expressive level of instability in both WS and WW (Figure 2), this does not bring greater adversities, since the popping expansion—which will be the most profitable trait in ISLA—is governed by oligogenes [50], not being as affected by environmental changes as the traits related to grain production. However, it is necessary to prospect this with some accuracy, since Kamphorst et al. [51] identified an average loss of 29.19% for PE between the WW and WS conditions in popcorn lines, although in our work this reduction was less prominent, of a magnitude of 3.5%.

In breeding programs, the discriminatory and representative capacity of a characteristic is extremely important, since it is possible to choose key characteristics for faster selection in addition to the possibility of obtaining gains in other characteristics by indirect selection. In this sense, in WS the traits PH, SPAD, GY and DM have the potential for greater discrimination and representativeness, favoring the selection of genotypes for a pool of characteristics of high interest for adaptation of germplasm to the water stress conditions. The grain yield is highly influenced by the production of dry matter by the plant [36] and, based on these results, the high discrimination of these traits indicates that selecting genotypes for the WS conditions based on DM will result in increases in GY.

The SPAD index is indicative of good functionality of plant leaf tissues [52]; therefore, its discriminatory power becomes important from the point of view of selection of superior genotypes for the condition of water stress [53]. It is essential to highlight that SPAD is a highly valuable feature for selection, as it is a fast, inexpensive and non-destructive method, in addition to allowing the generation of data before harvest, thus also favoring the acceleration of the breeding program. CE in WS was not representative, but has a discriminant potential. This fact indicates that the practice of selecting genotypes based on this trait does not point to concomitant increases in relation to the others; however, it is an efficient character to discriminate the genotypes studied in this condition.

In the WW condition, DM, GY and PH were the most discriminating and representative, and behavior similar to the WS condition can be observed. The traits 100GW, EL and NGR provided greater discrimination, but were not very representative. Conversely, SPAD was not very discriminating and unrepresentative. This inexpressive valuation for SPAD in WW may be associated with the fact that under optimal conditions of irrigation, plants tend to maintain values close to the chlorophyll content, and it is not possible to differentiate them based on this characteristic, and thus the existing variation between plants does not become expressive. Previous studies claim that stressful water environments provide greater expression of the heterogeneity of populations, when compared to normal conditions [54]. An even more classic example is the work of [55], who, when assessing the tolerance of four cotton genotypes to water stress, noticed that under conditions of full irrigation these genotypes did not differ as to the SPAD.

In our study, the genotypes that came closest to the ideotype for WS were BOZM260, 880POP and 288POP; and for WW were 880POP, BOZM260 and 288POP. Of these, two had already been appointed to form a compound, namely: 880POP and 288POP. Considering the results of the genotypes’ ranking, the BOZM260 genotype can be inserted, due to its good performance, especially in the WS condition. Selection based on traits and the creation of an ideotype is an effective strategy, since the analysis of the variance of the traits in WW and WS conditions, as well as their interaction with the environment, can point out key traits such as new criteria for selecting superior genotypes [41,56,57].

According to Yan [25], the genotype ranking analysis can provide an adequate estimate of the yield of each genotype. For this same author, in the evaluation of 33 different genotypes of wheat in eight environments, the correlation between the real yield and the estimates by the biplot method was 0.98. The efficiency of this analysis has already been confirmed in the selection of tomato genotypes [58], potatoes [59], barley [60], peppers [61] and, more recently, in cowpea [62].

## 4. Materials and Methods

### 4.1. Plant Material

Fifteen populations of popcorn belonging to the Germplasm Bank of the North Fluminense Darcy Ribeiro State University (UENF) were evaluated in two conditions of water availability (Table 2).

### 4.2. Experimental Design, Cultural Practices and Environmental Conditions

The experiment was carried out in the period of lowest rainfall for the North Fluminense region, from May to September 2018, at the Experimental Station of the Colégio Estadual Agrícola Antônio Sarlo, in Campos dos Goytacazes, Rio de Janeiro, Brazil (21°42′48″ S, 41°20′38″ W, and 17 m above sea level).

The experimental design was randomized complete blocks with three replications, under two irrigation regimes: well-irrigated (WW) and water stress (WS). Each plot consisted of a 4.80 m line, spaced 0.20 m between plants and 0.80 m between lines, with a total of 23 plants per line (62,500 plants ha^−1^). The cultural practices were made according to the recommendations for the culture.

The irrigation was applied with a drip system (flow rate 2.3 mm h^−1^), with one dripper per plant. To guarantee the imposition of water stress, the irrigation was suspended 10 days before male flowering in the WS condition. The irrigation system was equipped with hydrometers, ensuring precision in the volume of water applied to each water regime.

**Figure 5:**
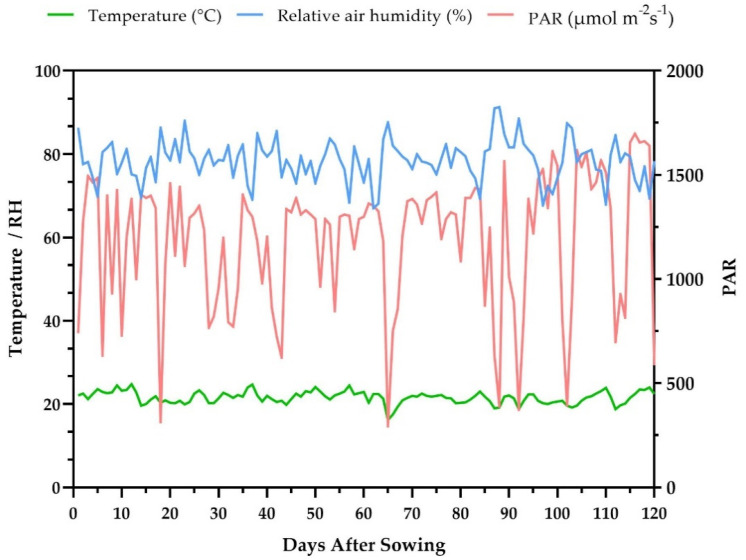
Values on days after sowing of temperature (°C), relative humidity (RH—%) and photosynthetically active radiation (PAR—µmol m^−2^s^−1^) throughout the growing period of the experiment with 15 popcorn populations under water stress (WS) and well-watered (WW) conditions; ; (-) signal indicating negative values.

An automatic station of the Brazilian Meteorological Institute (INMET) [63], located 100 m from the experiment, recorded the experimental conditions throughout the period of growth and development of the culture, with temperature variations being recorded (15.90–25.40 °C, average of 22 °C), relative humidity of the air (66.50–92.00%, average of 77%) and the average daily photosynthetically active radiation (PAR—1190 µmol m^−2^ s^−1^) (Figure 5).

Regarding the availability of water in the experiment, the WW condition received 187.89 mm, while in the WS condition the quantity of 69.30 mm was provided, in addition to the 148.20 mm precipitation recorded during the period (Table 3).

**Figure 6:**
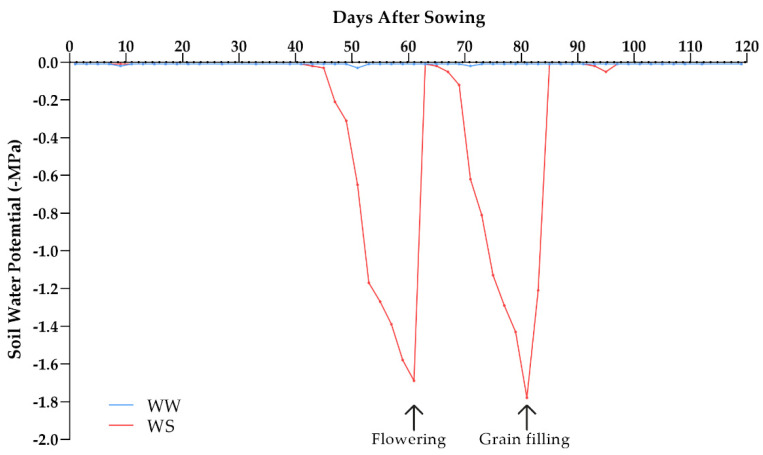
Soil water potential (−MPa) of the experiment with 15 popcorn populations under water stress (WS) and well-watered (WW) conditions.

The water potential of the soil was monitored with Decagon MPS-6 tensiometers (Decagon, Pullman, WA, USA) installed between plants, at a depth of 0.20 m. The soil in WW conditions was maintained at field capacity (−0.01 MPa), while under WS, the soil reached the permanent wilt point (−1.5 MPa) twice—the first time during flowering (59 days after sowing) and the second during grain filling (81 days after sowing) (Figure 6).

### 4.3. Phenotyping

The traits evaluated in the plant were: plant height (PH), relative chlorophyll content (SPAD), tassel length (TL) and number of tassel branches (NTB). PH was measured based on the distance between the plant′s insertion in the soil and the flag leaf, with the aid of a tape measure. The SPAD value was collected during the grain filling period (107 DAS), since this is the most harmful period of deficit in plants [35].

SPAD measurements were obtained weekly using a chlorophyll meter (SPAD 502, Minolta Co. Ltd., Osaka, Japan). For that, three locations were identified on the flag leaf (base, middle and apex) and three measures were taken in each location of the leaf [57], In order to minimize variations in the values obtained due to fluctuations in light intensity, it was adopted as a standardization to obtain these measurements between 10 and 12 h during the entire evaluation period. The TL was measured from the insertion in the flag leaf to the apex of the main branch, with the aid of a graduated ruler. NTB, on the other hand, was measured based on the count of these structures.

The harvest took place approximately 120 days after sowing. After the harvest, the shoot dry matter (DM) was estimated based on the collection of ten plants per plot, taken to the circulation oven for 72 h at 80 °C. After drying, the samples were weighed and, finally, the average values considered. The ear length (EL) was measured based on five ear samples per experimental plot, expressed in cm and obtained by means of a digital caliper. The number of rows of grains (NRG) and number of grains per row (NGR) were determined by counting them. The mass of one hundred grains (100GW) was determined by weighing (g) five samples per plot of 100 grains each.

The grain yield (GY) was corrected to 13% moisture and expressed in Kg ha^−1^. The popping expansion (PE) was measured for the mass of 30 g of grains, irradiated in a microwave oven, in a special paper bag for popping, in the power of 1000 W and 2.4 GHz, for two minutes and fifteen seconds. The volume of popcorn was quantified in a 2000 mL beaker. The PE was determined by the ratio of the volume obtained from popcorn and grain mass, expressed in mL g^−1^.

The traits related of root architecture were: support root angle (SRA) and crown root angle (CRA), which were obtained with the aid of a degree transferor and expressed in degrees (°) in relation to the ground; number of support roots (NSR), number of crown roots (NCR) and density of crown roots (DCR). For that, we used the methodology proposed by Trachsel et al. [64]. After harvesting, the soil of the water stress and control conditions received irrigation of 50 mm of water, facilitating the removal of the plants. Then, the root system of two plants per plot in both conditions was removed in soil cylinders 40 cm in diameter and 25 cm deep. Immediately after removal, these cylinders were washed for the complete removal of the soil and, thus, the characteristics SRA, CRA, NSR, NCR and DCR were measured.

### 4.4. Data Analysis

Individual analysis of variance (ANOVA) was performed for each experiment separately to verify the uniformity of the variance residue, followed by joint ANOVA, considering water availability (WW and WS) and genotype as fixed effects (*p* < 0.05). These analyzes were performed with the aid of the Genes software [65].

The multivariate analyzes of “which won where/what”, “means vs. stability”, “discriminativeness vs. representativeness” and “ranking genotypes” were performed considering all the characteristics in the genotype-by-trait (GT) biplot model [20], using the standardized values of the variables: GY, PE, EL, NGR, 100GW, SPAD, PH and DM. To generate the GT biplot graph, RStudio software was used [66]—package GGEbiplotGUI [17].

## 5. Conclusions

In the WS condition, the population BOZM260 stood out once, in general, presented the highest means for the evaluated traits, but 880POP was the most stable in low water availability conditions and, in WW, the 880POP population was the one with the highest mean.

The most representative and discriminating traits in the two conditions were GY and DM, while SPAD allowed reliable discrimination in the WS condition, which points to the successful selection of superior genotypes for drought conditions based on this non-destructive and easy-to-measure characteristic.

On basis of the analysis of the ideotype, we indicate the population 880POP as being superior for the condition of water limitation for both GY and PE, making it promising for the advancement of the popcorn breeding program in registering cultivars tolerant to drought conditions.

In addition, we indicate the formation of a compound consisting of the populations 574POP, 880POP, BOZM260 and ISLA for the extraction of lines and obtaining hybrids and/or the implementation of a recurrent selection program. This is because, even though they are not superior for the whole set of characteristics, these populations presented high values for the main characteristics of interest in WS conditions (PE, RG, NGR and DM) indicating great tolerance to water scarcity in soil.

Due to the genetic variability found among the 15 Latin American populations of popcorn under the irrigated and water stress conditions, these populations are considered of interest for obtaining superior inbred lines for drought tolerance and/or efficiency in the use of water. This study is going to allow the establishment of a collection of great importance to maize germplasm and to provide information to facilitate the process of selection in the breeding programs focused on drought tolerance.

## Figures and Tables

**Table 1:** Summary of individual and joint ANOVA, means, standard deviations and coefficient of variation (CV%) of morpho-agronomic, physiological and root traits of 15 populations of popcorn evaluated under water stress (WS) and well-watered (WW) conditions.

Trait	Water Regime (A)	Mean Squares		Mean ± SD	CV (%)
		Genotype (G)	G × A		
		(DF = 14)	(DF = 14)		
SPAD	WW	73.84 ^ns^	109.19 **	33.25 ± 6.44	19.35
	WS	159.92 **		28.02 ± 5.49	19.60
PH	WW	1074.06 **	192.25 ^ns^	181.55 ± 12.43	6.85
	WS	888.84 **		163.48 ± 9.32	5.70
TL	WW	7.63 **	4.58 **	12.04 ± 0.42	3.51
	WS	8.20 **		11.95 ± 0.58	4.88
NTB	WW	14.26 **	7.33 **	19.31 ± 2.06	5.97
	WS	9.74 **		16.02 ± 1.15	12.85
EL	WW	4.56 **	2.55 **	12.79 ± 0.86	6.73
	WS	5.62 **		11.30 ± 1.02	8.99
NRG	WW	4.28 **	1.43 ^ns^	13.24 ± 0.94	7.07
	WS	4.79 ^ns^		12.64 ± 1.59	12.58
NGR	WW	22.16 **	28.22 *	27.41 ± 2.84	10.36
	WS	60.97 **		23.24 ± 4.10	17.63
100GW	WW	12.33 **	2.01 ^ns^	15.99 ± 1.33	8.29
	WS	25.49 **		15.07 ± 1.15	7.65
GY	WW	1,776,800.80 **	288,045.29 **	2684.28 ± 349.06	13.00
	WS	760,443.71 **		1862.62 ± 254.88	13.68
PE	WW	207.50 **	5.48 ^ns^	20.87 ± 2.04	9.77
	WS	166.59 **		20.14 ± 1.63	8.08
DM	WW	8706.63 **	3392.40 **	201.69 ± 32.48	16.11
	WS	6384.97 **		191.49 ± 32.07	16.75
SRA	WW	147.95 **	1.12 ^ns^	61.24 ± 2.64	4.32
	WS	139.86 **		60.87 ± 2.38	3.90
CRA	WW	49.34 ^ns^	10.73 ^ns^	68.76 ± 6.69	9.73
	WS	93.51 **		67.71 ± 2.44	3.60
NSR	WW	16.47 **	1.10 ^ns^	12.96 ± 0.99	7.62
	WS	14.80 **		13.20 ± 1.93	14.65
NCR	WW	60.90 **	2.53 ^ns^	20.16 ± 0.78	3.88
	WS	55.45 **		19.91 ± 1.50	7.51
DCR	WW	0.93 **	1.54 **	4.32 ± 0.27	6.25
	WS	2.34 **		5.20 ± 0.31	5.87

SPAD—relative chlorophyll content; PH—plant height (cm); TL—tassel length (cm); NTB—number of tassel branches; EL—ear length (cm); NRG—number of rows of grains (un); NGR—number of grains per row (un); 100GW—100 grains weight (g); GY—grain yield (Kg ha^−1^); PE—popping expansion (g mL^−1^); DM—shoot dry matter (g); SRA—support root angle (°); CRA—crown root angle (°); NSR—number of support roots (un); NCR—number of crown roots (un); e DRC—density of crown roots. * and ** indicate significance and ^ns^ indicate not significant, at 5 and 1% of probability, respectively, by Test F.

**Table 2:** Information about accession ID, origin, donor institution and climate adaptation of the fifteen Latin-American populations used in the experiment.

Accession ID		Origin	Donor Institution	Climate Adaptation
1	288POP	Guaraciaba/SC, Brazil	-	Subtropical
2	574POP	Guaraciaba/SC, Brazil	-	Subtropical
3	880POP	Guaraciaba/SC, Brazil	-	Subtropical
4	ARZM13050	Argentina, Brazil	CIMMYT	Temperate/Tropical
5	BARÃOUFV	Viçosa/MG, Brazil	UFV	Temperate/Tropical
6	BOYA462	Colombia	CIMMYT	Temperate/Tropical
7	BOZM260	Bolívia	CIMMYT	Temperate/Tropical
8	BRS Angela	Sete Lagoas/MG, Brazil	Embrapa	Tropical
9	CHZM13134	Chile	CIMMYT	Temperate/Tropical
10	ISLA	Paraná	ISLA S/A	Temperate/Tropical
11	PARA172	Paraguay	CIMMYT	Temperate/Tropical
12	UNB2-C0	Campos dos Goytacazes/RJ, Brazil	UENF	Tropical
13	UNB2-C6	Campos dos Goytacazes/RJ, Brazil	UENF	Tropical
14	UNB2-C8	Campos dos Goytacazes/RJ, Brazil	UENF	Tropical
15	URUG298A	Uruguay	CIMMYT	Temperate/Tropical

RJ—Rio de Janeiro; MG—Minas Gerais; SC—Santa Catarina; UFV—Universidade Federal de Viçosa; CIMMYT—International Maize and Wheat Improvement Center; UENF—Universidade Estadual do Norte Fluminense.

**Table 3:** Weekly precipitation and irrigation (mm) applied in the well-watered (WW) and under water stress (WS) conditions.

Weeks after Sowing	Rainfall	Amount of Water (mm)			
		Well-Watered		Water Stress	
		Irrigation	Total	Irrigation	Total
1	17.00	7.21	23.20	6.20	23.20
2	6.00	10.97	16.97	10.24	16.24
3	0.00	10.13	10.13	9.86	9.86
4	10.60	10.72	21.32	10.27	20.87
5	5.20	8.35	13.55	8.43	13.63
6	2.00	11.60	13.60	12.18	14.18
7	0.00	12.94	12.94	12.12	12.12
8	0.00	10.86	10.86	-	0.00
9	0.00	18.79	18.79	-	0.00
10	0.00	18.95	18.95	-	0.00
11	30.80	1.14	31.94	-	30.80
12	0.00	16.73	16.73	-	0.00
13	0.00	14.00	14.00	-	0.00
14	65.00	2.00	67.00	-	65.00
15	0.00	13.50	13.50	-	0.00
16	9.20	10.00	19.20	-	9.20
17	2.40	10.00	12.40	-	2.40
Total	148.20	187.89	335.08	69.30	217.50
